# Resting-State Activity Changes Induced by tDCS in MS Patients and Healthy Controls: A Simultaneous tDCS rs-fMRI Study

**DOI:** 10.3390/bioengineering12060672

**Published:** 2025-06-19

**Authors:** Marco Muccio, Giuseppina Pilloni, Lillian Walton Masters, Peidong He, Lauren Krupp, Abhishek Datta, Marom Bikson, Leigh Charvet, Yulin Ge

**Affiliations:** 1 Department of Radiology, NYU Grossman School of Medicine, New York, NY 10016, USA; 2 Department of Neurology, NYU Grossman School of Medicine, New York, NY 10016, USA; 3 Research and Development, Soterix Medical, Inc., Woodbridge, NJ 07095, USA; 4 Department of Biomedical Engineering, City College of New York, New York, NY 10031, USA

**Keywords:** transcranial direct current stimulation (tDCS), multiple sclerosis (MS), healthy controls, resting-state functional MRI (rs-fMRI), neuromodulation, fractional amplitude of low-frequency fluctuations (fALFFs)

## Abstract

Transcranial direct current stimulation (tDCS) is a safe, well-tolerated method of non-invasively eliciting cortical neuromodulation. It has gained recent interest, especially for its positive clinical outcomes in neurodegenerative diseases such as multiple sclerosis (MS). However, its simultaneous (during tDCS) and cumulative effects (following repeated tDCS sessions) on the regional brain activity during rest need further investigation, especially in MS. This study aims to elucidate tDCS’ underpinnings, alongside its therapeutic impact in MS patients, using concurrent tDCS-MRI methods. In total, 20 MS patients (age = 48 ± 12 years; 8 males) and 28 healthy controls (HCs; age = 36 ± 15 years; 12 males) were recruited. They participated in a tDCS-MRI session, during which resting-state functional MRI (rs-fMRI) was used to measure the levels of the fractional amplitude of low-frequency fluctuations (fALFFs), which is an index of regional neuronal activity, before and during left anodal dorsolateral prefrontal cortex (DLPFC) tDCS (2.0 mA for 15 min). MS patients were then asked to return for an identical tDCS-MRI visit (follow-up) after 20 identical at-home tDCS sessions. Simultaneous tDCS-induced changes in fALFF are seen across cortical and subcortical areas in both HC and MS patients, with some regions showing increased and others decreased brain activity. In HCs, fALFF increased in the right pre- and post-central gyrus whilst it decreased in subcortical regions. Conversely, MS patients initially displayed increases in more posterior cortical regions but decreases in the superior and temporal cortical regions. At follow-up, MS patients showed reversed patterns, emphasizing significant cumulative effects of tDCS treatment upon brain excitation. Such long-lasting changes are further supported by greater pre-tDCS fALFFs measured at follow-up compared to baseline, especially around the cuneus. The results were significant after correcting for multiple comparisons (p-FDR < 0.05). Our study shows that tDCS has both simultaneous and cumulative effects on neuronal activity measured with rs-fMRI, especially involving major brain areas distant from the site of stimulation, and it is responsible for fatigue and cognitive and motor skills.

## 1. Introduction

Transcranial direct current stimulation (tDCS) is a well-tolerated method of safely and, most importantly, non-invasively eliciting brain activity via the application of weak electrical currents [1,2,3]. Its applications have been extensively investigated in a range of neurological and neurodegenerative conditions [4,5,6,7], often as treatment in conjunction with other rehabilitation methods, such as cognitive training [8,9,10]. However, most of these studies focused on the clinical aspects and outcomes of tDCS, leaving a gap in knowledge of the corresponding neuronal underpinnings. Recent technological advancements have allowed tDCS to be safely applied using an MRI scanner, which is also known as concurrent tDCS-MRI, therefore enabling the simultaneous acquisition of imaging measures, such as brain activity fluctuations, during the stimulation itself.

A neurodegenerative disease that has received increasing attention in relation to tDCS-related research is multiple sclerosis (MS). This is a chronic, immune-mediated disorder of the central nervous system characterized by widespread inflammation often leading to progressive demyelination, axonal injury, and ultimately neurodegeneration [11]. Such disease-linked effects then appear as characteristic, hypertense MRI lesions in the white-matter regions of the brain and spinal cord.

Axonal demyelination in white matter is often a cause of neuronal loss and, therefore, gray-matter atrophy, which contribute to the clinical aspects of MS. This aspect is now recognized as a major driver of disability progression at the motor and cognitive level in MS. This condition is highly varied in its onset, presentation, and progression, but it typically begins in younger adulthood and might involve periods of spontaneous remission–relapse (relapsing–remitting MS) and would inevitably progress into progressive degeneration (secondary progressive MS) [12,13].

Interventional tDCS studies have already shown that tDCS has significant effects in patients with MS, reporting a reduction in the common symptoms of fatigue [14], improvements in motor [15] and cognitive [16] functions, and greater neuronal responses to stimulation compared to only motor or cognitive training treatments [17,18,19]. Physiologically, studies have also reported increases in regional and global cerebral blood flow and neuronal activity [20,21,22], providing further evidence of the direct cerebrovascular and neuronal effects of tDCS.

In addition, attention has recently turned to understanding the cumulative effects of multiple tDCS treatment sessions on brain metabolic properties, with several studies reporting greater and long-lasting metabolic changes following multiple stimulation sessions compared to single applications [23,24,25,26], even in MS patients [22].

While these findings highlight the global cerebrovascular and metabolic effects of tDCS, recent imaging studies have started exploring its impact on brain activity and connectivity through the concurrent acquisition of resting-state functional MRI (rs-fMRI) during tDCS. These studies have reported significant perturbations and modulations in brain activity [27,28] and functional connectivity [29,30,31,32], which extends beyond the areas of electrode placement.

In the present study, we used rs-fMRI to examine regional brain activity changes resulting from tDCS applications in patients with MS and healthy controls (HCs) during stimulation (simultaneous effects). Moreover, we employed a remotely supervised at-home tDCS treatment protocol [14] to investigate the cumulative effects of repeated stimulation, measured at follow-up in MS patients (cumulative effects). The use of simultaneous tDCS-MRI represents a key advancement because, as opposed to commonly used offline setups, it allows for a reduction in the wash-out effects of the stimulation.

Using this novel approach, this study therefore addresses the significant need for a deeper understanding of simultaneous and cumulative tDCS-induced changes in regional brain activity, which will be fundamental to evaluate its therapeutic potential in neurodegeneration and optimizing its clinical use. Our analysis focuses on the voxel-based changes in resting-state neuronal activity, measured by the fractional amplitude of low-frequency fluctuations (fALFFs). This is a commonly used method that links the regional changes in blood oxygenation and, therefore, MRI signal changes to neuronal activity based on the widely accepted concept of neurovascular coupling. FALFF has also been previously used to measure neuronal activity at rest in healthy and diseased populations [33,34,35]. Based on the known biophysiological effects of tDCS, we hypothesized that tDCS would lead to increased fALFF signals during stimulation compared to pre-stimulation in both healthy controls and MS participants, aligning with the previously reported global neuromodulatory effects of tDCS [20,21,22], with potentially stronger modulation in healthy individuals. Additionally, we expected that a greater number of brain regions would show tDCS-related fALFF changes in MS participants at follow-up relative to the baseline, reflecting cumulative or delayed stimulation effects. Finally, we anticipated higher pre-stimulation fALFF levels at follow-up compared to baseline as a possible indicator of sustained neuromodulatory impact following repeated tDCS sessions.

## 2. Methods

### 2.1. Participants

Study participants were recruited from the NYU Langone Health MS Comprehensive Care Center, the National MS Society, and other local community referrals. A phone screening process allowed the exclusion of any participant with a history of head injuries; neurological diseases (for healthy controls); non-MRI-safe implants; MS diagnosis confirmation (any subtype) scored on the basis of the expanded disability status scale (EDSS); and any contraindications to tDCS, such as skin disorders or defects (e.g., rashes, eczema) and current seizure disorders. MS patients were further screened for any other neurological comorbidities, such as traumatic brain injury, epilepsy, and tumors.

The final sample size used for this prospective observational study was 20 MS patients (age = 48 ± 12 years; 8 males) and 28 HCs (age = 36 ± 15 years; 12 males). IRB approval was provided by NYU Langone Health as part of a larger clinical study (study identifier: S18-005548).

### 2.2. Study Design and Imaging Acquisition

All participants were asked to partake in a baseline concurrent tDCS-MRI session, and MS patients were then asked to return for a follow-up tDCS-MRI visit. Between the two visits, patients received an at-home, remotely supervised treatment composed of 20 tDCS sessions, which had the same stimulation parameters used in the scanning sessions. Stimulation delivery and quality were tracked by remotely checking the electrode contact quality of the minimum impedance of 8/10.

Each tDCS-MRI visit was composed of a ‘pre-tDCS’ phase (15–20 min), during which no stimulation was given to the participant, and a ‘during-tDCS’ phase (15 min), during which 2.0 mA tDCS was given to the participant.

**Figure 1:**
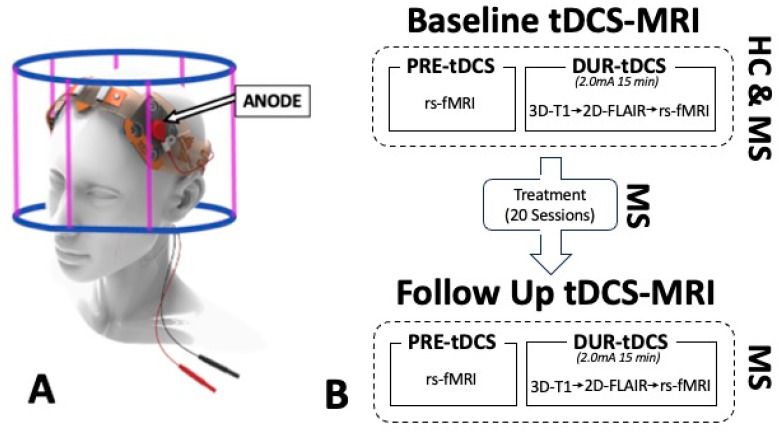
(**A**) Transcranial direct current stimulation (tDCS) setup with an anodal left dorsolateral prefrontal cortex (DLPFC; F3) montage within the 3T MRI scanner. (**B**) Study design showing resting-state fMRI (rs-fMRI) data acquisition at baseline for healthy controls (HCs) and multiple sclerosis (MS) patients and at follow-up (MS only) after undergoing a treatment of 20 at-home tDCS sessions (MS only).

Stimulation was delivered using an MRI-compatible tDCS device (1 × 1 tDCS Model 1300 Low-Intensity Stimulator, Soterix Medical Inc., Woodbridge, NJ, USA) via the application of two conductive rubber electrodes (5 × 5 cm each) soaked in saline to reduce resistivity, which were placed over the forehead of the participant with a typical left dorsolateral prefrontal cortex (DLPFC) montage (anode on F3, cathode on F4; Figure 1A). The current intensity chosen for the stimulation was 2.0 mA, which was previously shown to successfully elicit and modulate neuronal activity [20,22,36,37], and was manually ramped up/down, ensuring adequate skin–electrode contact quality (impedance of 8 out of a maximum of 10 kohms). The ramp-up and ramp-down process only lasted a few seconds, and no imaging measure was acquired during these specific times.

Imaging data were acquired using a 3T MRI scanner (Prisma, Siemens, Munich, Germany) fitted with a 64-channel head coil. Anatomical images were acquired at the beginning of the during-tDCS phase to provide enough time for the stimulation to penetrate the skull and reach the brain tissue. Such routine MRI sequences were as follows: 3D-T1 magnetization-prepared rapid acquisition gradient echo (MPRAGE; acquisition time—TA  =  4 min 19 s; repetition time—TR  =  2300 ms; echo time—TE  =  2.98 ms; flip angle—FA  =  9 degrees; spatial resolution  =  1 × 1 × 1 mm) and 2D oblique axial fluid-attenuated inversion recovery (FLAIR; TA  =  2 min 44 s; TR  =  9000 ms; TE  =  2.98 ms; FA  =  150 degrees; spatial resolution  =  0.7 × 0.7 × 2 mm). In each phase and each MRI visit, the same resting-state functional MRI (rs-fMRI) sequence, with identical scanning parameters, was applied to allow an adequate comparison of measurements between visits, timepoints, and groups. During rs-fMRI image acquisition, the subjects were instructed to stay still with their eyes closed but without falling asleep, and the sequence parameters used were the following: TR = 2000 ms; TE = 25 ms; spatial resolution = 3.0 × 3.0 × 3.0 mm; FA = 70 deg; number of slices = 35; distance factor = 20%; timepoints = 153; acquisition = interleaved. The comprehensive study design is represented in Figure 1B.

### 2.3. Imaging Processing and Analysis

**Figure 2:**
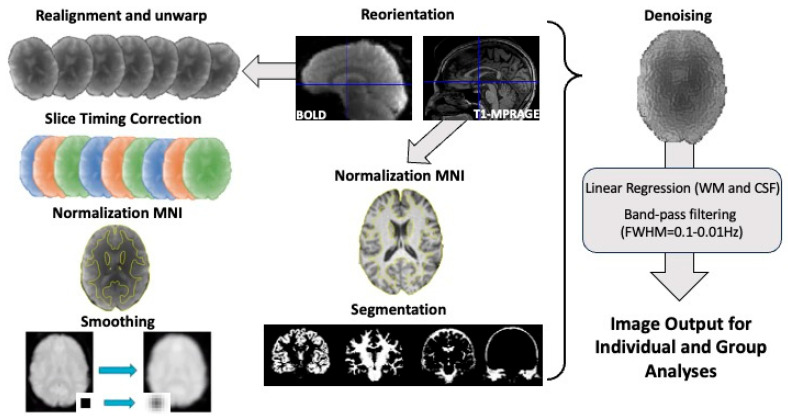
Preprocessing pipeline used to normalize and standardize the structural and functional datasets of both healthy controls (HCs) and multiple sclerosis (MS) patients before undergoing subject-level and group-level statistical analyses.

Several steps were taken to obtain the results presented in this study. From preprocessing, MRI images were obtained for denoising, quality checks, and, finally, individual- and group-level analysis. All these steps are described in more detail in the sections below and are represented in Figure 2.

#### 2.3.1. Preprocessing

The results included in this manuscript come from analyses performed using CONNtoolbox (www.nitrc.org/projects/conn) release 22.a [38] and SPM12 (Wellcome Trust Center for Neuroimaging, Institute of Neurology, London, UK; https://www.fil.ion.ucl.ac.uk/spm/software/spm12/) implemented in MATLAB R2022a (Mathworks, Natick, MA, USA; https://www.mathworks.com/products/matlab.html).

Functional and anatomical data were firstly preprocessed using a default preprocessing pipeline [39,40]. These steps included the following: (1) functional realignment with corrections of susceptibility-induced distortion interactions, which realigned multiple time-point images of a functional dataset by coregistering all of the images to a reference image (first image) via the least-squares approach and a 6-parameter (rigid body) transformation; (2) slice timing correction, correcting for temporal misalignments between different slices of the functional dataset by resampling each slice’s BOLD time series to a common mid-acquisition time (T2/2); (3) direct coregistration of the functional images to the corresponding structural ones via rigid body transformation; (4) direct segmentation of structural images into gray-matter (GM), white-matter (WM), and CSF-tissue classes using an SPM-unified segmentation algorithm [41,42] with a default tissue probability map template; (5) direct normalization of both structural and functional images into the Montreal Neurological Institute (MNI); lastly, (6) functionally preprocessed images were smoothed using spatial convolution with a 6 mm full-width half-maximum (FWHM) Gaussian kernel to reduce partial volume effects.

To ensure appropriate image and data quality, each preprocessing step was manually checked for image distortions or artifacts. In addition, images reporting head motion along (>1 voxel) or around (>0.5 degrees) the main axis were removed. In our sample size of 20 patients, no subjects were excluded due to poor image quality.

#### 2.3.2. Denoising

Before conducting voxel-based, single-subject statistical analysis, a correction for physiological noise was carried out using the in-built denoising method [43]. This was achieved by regressing potential confounding effects, such as the following: motion parameters (along and around the x, y, and z axes) and their first-order derivatives (12 factors in total); outlier scans detected in slice time corrections (below 23 factors; otherwise, the subject is excluded); linear trends measured in the WM and CSF regions (2 factors) within each functional run; and finally, the bandpass frequency filtering of the BOLD time series (0.01 Hz and 0.1 Hz) to remove cardiac and respiratory-induced noise.

#### 2.3.3. Individual- and Group-Level Analyses

Fractional amplitude of low-frequency fluctuation (fALFF) maps characterizing low-frequency BOLD signal variability at each voxel were estimated as the ratio between the root mean square (RMS) of the BOLD signal after denoising divided by the same measure computed before band-pass filtering [44,45]. The FALFF measures across voxels were then rank, sorted, and normalized separately for each individual subject and session (pre- and during-tDCS) using a Gaussian inverse cumulative distribution function with zero mean and unit variance.

Group-level analyses were performed using a General Linear Model (GLM). For each individual voxel, a separate GLM was estimated, with first-level connectivity measures at this voxel as dependent variables (one independent sample per subject and one measurement per scanning phase) and groups or other subject-level identifiers as independent variables. Voxel-level hypotheses were evaluated using multivariate parametric statistics with random effects across subjects and sample covariance estimation across multiple measurements. Inferences were performed at the level of individual clusters (groups of contiguous voxels). Cluster-level inferences were based on parametric statistics from Gaussian random field theory. A statistical threshold was applied using a combination of a *p* < 0.001 cluster-forming voxel-level threshold and a false discovery rate (FDR)-corrected p-FDR of <0.05 with cluster-size thresholds [46].

## 3. Results

The rs-fMRI analysis, based on fALFF measurements, revealed several brain regions of increased and decreased activity as a result of tDCS (simultaneous effects) in both the healthy and MS groups.

In healthy subjects, we observed significant fALFF differences (cluster p-FDR < 0.05) between pre- and during-tDCS in nine major clusters. Of these, six clusters (3829 voxels in total) showed a significant increase in brain activity, whilst three (5910 voxels in total) reported a significant fALFF decrease. The clusters’ details for both increased and decreased fALFF are summarized in Table 1, together with a list of the major brain cortical and subcortical regions in which such voxels are observed.

**Figure 3:**
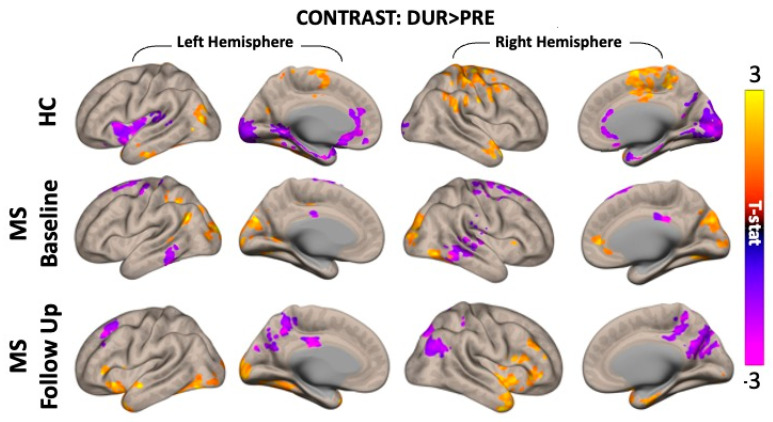
Group-level analyses results showing clusters of voxels with significant differences between fALFF measures during-tDCS compared to pre-tDCS in healthy controls (HCs) and multiple sclerosis (MS) patients at baseline and follow-up visits. Notice how, especially in the cortical areas, fALFF seems to be greater during tDCS compared to pre-tDCS (yellow voxels), whilst in more subcortical areas, fALFF measures appear to be decreased from pre- to during-tDCS. The statistical threshold was set to p-voxel < 0.001 and p-cluster < 0.05 and corrected for multiple comparisons using the false discovery rate (FDR). Warmer tones in the color scale indicate an increase in fALFF during tDCS compared to pre-tDCS, whilst colder tones indicate the opposite: a decrease in fALFF.

In MS patients, in both visits, several clusters of voxels have also reported significant changes (cluster p-FDR < 0.05) in fALFF as a result of tDCS. At baseline, six major clusters (2653 voxels in total) showed a significant increase in brain activity, and seven different clusters (2401 voxels in total) showed a significant decrease in fALFF. At follow-up, however, we observed nine major clusters (2532 voxels in total) with increased fALFF due to tDCS and five clusters (1721 voxels in total) showing a significant decrease in fALFF. More details for each cluster and the overall brain regions showing such changes in brain activity are shown in Table 2 (baseline) and Table 3 (follow-up). Both HC and MS results for simultaneous tDCS effects are represented in Figure 3.

Statistical analyses comparing the before-and-after treatment changes in brain activity were then carried out to investigate the cumulative effects of repeated tDCS sessions. Only three clusters (1171 voxels in total) showed a significant increase in brain activity when comparing pre-tDCS fALFF levels at follow-up versus pre-tDCS at baseline: cluster 1 mainly involving the bilateral cuneal cortex; cluster 2 including parts of the right lateral occipital cortex; and cluster 3 involving the right post- and precentral gyri. Significant decreases in pre-tDCS levels between sessions were observed in only one cluster (385 voxels), involving the major right frontal brain regions such as the frontal pole and middle and inferior frontal gyri.

**Figure 4:**
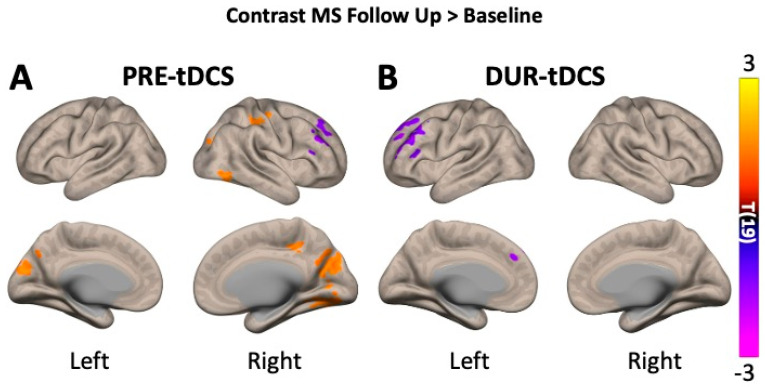
Group-level analyses addressing the cumulative effects of tDCS by comparing fALFF measures in multiple sclerosis (MS) patients at baseline versus follow-up at pre-tDCS (**A**) and during-tDCS (**B**). Notice how, at follow-up, most of the voxel clusters show increased brain activity (fALFF; orange voxels) in pre-tDCS compared to baseline measures, even before re-introducing the stimulation. The statistical threshold was set to p-voxel < 0.001 and p-cluster < 0.05 and corrected for multiple comparisons using the false discovery rate (FDR). Warmer tones in the color scale indicate an increase in fALFF in follow-up compared to baseline, whilst colder tones indicate the opposite: a decrease in fALFF.

Comparisons of during-tDCS levels between baseline and follow-up (Figure 4) only showed decreased fALFF in two major clusters (732 voxels in total), which was mainly present in the left brain regions of the frontal lobe: frontal pole and superior, middle, and inferior frontal gyri. The results of the cumulative effect analysis are grouped in Table 4.

**Figure 5:**
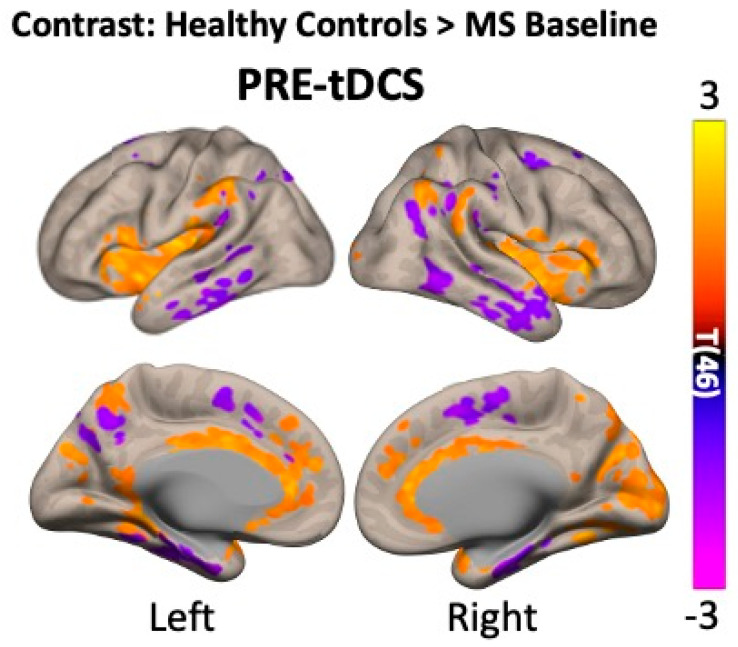
Group-level analyses addressing the potential disease-linked differences in baseline resting-state activity by comparing pre-tDCS fALFF measures in multiple sclerosis (MS) patients to healthy controls (HCs). Notice how HCs seem to have greater resting-state activity, especially in subcortical areas (orange voxels), whilst the opposite can be seen in more cortical areas (purple clusters). The statistical threshold was set to p-voxel < 0.001 and p-cluster < 0.05 and corrected for multiple comparisons using the false discovery rate (FDR). Warmer tones in the color scale indicate greater fALFF in MS patients compared to healthy controls, whilst colder tones indicate the opposite: a decrease in fALFF.

Finally, a statistical comparison between healthy controls and MS patients at baseline (Figure 5) was carried out to address any disease-related differences in resting-state brain activity. Several voxel clusters showed differences between the two groups in pre-tDCS, and these results are summarized in Table 5.

## 4. Discussion

Recently, tDCS has received growing interest, especially due to the several reports of clinical improvement in many neurological and neurodegenerative diseases [47,48,49]. In particular, in MS, tDCS has been observed to reduce fatigue and improve motor skills, especially when applications were repeated over several sessions [50]. In this manuscript, we report significant changes in brain activity as a result of the tDCS treatment in MS subjects and HCs by utilizing concurrent tDCS and rs-fMRI. Our results go beyond demonstrating that tDCS induced a neuronal response in MS subjects, which is still supported only by a few other studies, by including the established long-lasting effects that are linked to repeated stimulation sessions. Moreover, we offer a significant, strong basis for the changes in brain activity in key areas that might be linked to behavioral and clinical changes, which will be investigated in future studies, and in this manuscript, we only speculate on the potential clinical implications that our imaging results might help explain.

### 4.1. Healthy Controls

In healthy controls, simultaneous tDCS treatment led to increased and decreased brain activity in several cortical and subcortical areas (Figure 3, top row). Most clusters showed increased fALFF in the right hemisphere. We initially hypothesized that activity would increase around the anode (left lateral frontal areas), but consistent with other rs-fMRI studies, we observed more widespread effects in both ipsilateral and contralateral regions [28,51]. The most prominent areas showing increased activity were the pre- and post-central gyri (motor and sensory), the temporal pole (memory), the supramarginal gyrus (empathy), and other parts (parts of the default mode network), suggesting potential cognitive and psychological changes [15,52,53].

Interestingly, activity was reduced in the anterior cingulate gyrus and brainstem, which are regions involved in regulating breathing, heart rate [54,55], and moods [56,57,58]. This reduction may reflect a more restful state during tDCS, aligning with previous findings on how stimulation affects neuronal excitability beyond the target area [59,60].

### 4.2. MS Patients

In MS patients, fewer immediate tDCS-induced changes were observed (Figure 3, middle row), indicating that a single application may not elicit a widespread response. However, significant increases were noted in areas related to goal-directed behavior, visual processing, and motor skills (e.g., cuneus, precuneus, occipital pole). On the other hand, decreases in activity were observed in somatosensory and memory-related regions (pre- and post-central gyri and temporal lobe).

At the follow-up visit (Figure 3, bottom row), after the 20-tDCS-session treatment, MS patients showed increased activity in the right frontal and temporal regions and decreased activity in the cingulate, occipital, and parietal lobes. These changes likely reflect long-term tDCS effects (Figure 4), with increased baseline neuronal activity between sessions, therefore impairing any further increase in neuronal activity. This suggests the possibility of a neuromodulation plateau, beyond which further tDCS applications may yield diminishing returns. The observed delayed or cumulative fALFF changes may reflect plasticity-related mechanisms, such as synaptic reorganization or long-term potentiation. While speculative, this aligns with the existing models of neuroplasticity following repeated stimulation. However, no direct clinical measures were included; thus, functional relevance remains to be tested in future research integrating behavioral assessments.

### 4.3. HC and MS

We then addressed the disease-linked differences in resting-state brain activity, comparing pre-tDCS measures in HCs to MS patients at baseline (Figure 5).

We found greater neuronal activity in the subcortical areas of HCs (cingulate gyrus, precuneus, and inferior frontal gyrus) and higher activity in the pre- and post-central gyri and temporal lobe in MS. These differences suggest that MS-related neurodegeneration may affect deeper brain structures whilst increasing activity in superficial areas as a compensatory mechanism. This may explain why we observed different tDCS responses in the cortical and subcortical regions between HC and MS.

While direct investigations into tDCS-induced regional brain activity in MS are limited, it has been shown that regional gray-matter atrophy correlates with disability in relapsing-remitting MS [61,62]. Our findings also support the hypothesis that altering neuronal firing in one area affects connected regions, as reported in several imaging studies [63,64]. Moreover, repeated tDCS could restore neuronal activity in diseased neurons, but further research is needed to support such a hypothesis and better assess structural changes over time. However, it must be stressed that the divergent patterns observed in MS versus healthy participants may reflect structural differences, such as brain atrophy and neuronal loss, or pathophysiological aspects, including lesion burden and distribution, for which their influence should be more systematically addressed in future studies.

This study, therefore, presents a novel approach by utilizing concurrent tDCS-MRI to directly measure the immediate and cumulative neuronal effects of tDCS. This innovative approach allows for the real-time monitoring of the brain activity changes induced by stimulation, providing unprecedented insights into the potential use of tDCS. For instance, the integration of imaging techniques such as rs-fMRI holds great potential for guiding advanced tDCS therapeutic techniques. For example, several studies have shown that functional connectivity measures could be used to predict responses to tDCS [65,66,67].

Moreover, the significant neuromodulation observed in this study, especially in MS patients, underscores the dynamic and potentially therapeutic role of tDCS in neuroplasticity. An important finding of our study is that repeated sessions of tDCS result in long-lasting effects, specifically causing the baseline neuronal activity of certain brain regions to remain elevated before reintroducing the stimulation. It could therefore be inferred that tDCS could lead to increased local neuroplasticity or the neuronal recovery of diseased neurons persisting beyond the stimulation phase.

Although no clinical or behavioral outcomes were measured in this study, the observed fALFF modulations may reflect the underlying mechanisms relevant to functional symptoms in MS, a possibility that warrants future investigation. Further preclinical and clinical studies are necessary to support this theory more reliably, and future studies with larger samples should explore how individual factors such as EDSS, disease duration, or age influence the neuromodulatory response to tDCS. However, our results set the basis for future studies investigating these key aspects, which might have important consequences in informing future tDCS therapeutic guidelines.

Besides the relatively small sample size of this study, other limitations must be acknowledged. In particular, the inclusion of a sham tDCS group could have strengthened the reliability of our results, and the lack of this group does not allow us to completely exclude the influence of nonspecific stimulation, such as a placebo effect. However, the use of this type of control group is still debated in the literature since it does not allow the effective blinding of subjects to the stimulation [68,69] and might also still have some bio-physiological effects on brain tissue polarization [70,71,72]. Although the absence of a sham stimulation control group limits the ability to infer causality, future studies should include a placebo condition to isolate the specific effects of tDCS from nonspecific or placebo-driven changes. In addition, other factors might have influenced our measurements, such as stimulation parameters (e.g., duration, current intensity, montage) [26,73,74,75,76,77], body positions [78], and cerebrovascular morphologies [79,80]. Finally. Although we have used fALFF to infer brain and neuronal activity properties in this manuscript, we must acknowledge that this is an indirect measure that actually looks at the oscillations of blood oxygenation in the brain and, therefore, is strongly affected by other physiological and cerebrovascular properties.

Further studies should expand on our results to investigate the neuronal effects of different electrode montages and current intensities, as well as the lingering after-effects of stimulation. Moreover, studies with larger sample sizes might investigate the potential of imaging markers, such as changes in brain activity, as a predictor of clinical responses to tDCS and tDCS-paired treatments.

## 5. Conclusions

In conclusion, the results presented in this manuscript demonstrate that tDCS successfully modulates neuronal activity in both healthy subjects and MS patients, although with different increase and decrease patterns over cortical and subcortical areas. Moreover, such acute responses to tDCS also hold cumulative properties, causing long-lasting changes in brain activity resulting from tDCS treatment. Our view is that concurrent tDCS and rs-fMRI hold important potential in correlating the clinical outcomes of innovative therapeutics, such as tDCS.

## Figures and Tables

**Table 1:** Simultaneous tDCS effects in healthy controls.

Main Brain Regions	Coordinates (x, y, z)	Voxels	Pre-tDCS (Mean; a.u.)	Dur-tDCS (Mean; a.u.)	Percentage Change (%)	T-Stat [27]	P-FDR
**Increased fALFF**							
Pre and Postcentral Gyri Right	‘+4 +0 +72’	1877	0.019201	0.368802	90.10288	9.66	<0.0001
Inferior Temporal Gyrus Right	‘−54 −16 −30’	1013	−0.32587	0.001911	98.8342	10.14	<0.0001
Precuneus Cortex	‘−38 −84 +20’	271	0.119524	0.443586	57.54851	6.76	0.016756
Temporal Pole Right	‘+48 +18 −32’	240	−0.42706	−0.05268	78.0369	7.25	0.027668
Supramarginal Gyrus Right	‘+60 −26 +28’	219	0.391473	0.707374	28.74843	7.18	0.03852
Middle Frontal Gyrus Right	‘+46 +12 +54’	209	−0.14105	0.231028	24.18118	6.76	0.042050
**Decreased fALFF**							
Brain Stem	‘−6 −40 +0’	3909	−0.23085	−0.66331	48.36413	−5.52	<0.0001
Occipital Pole Bilateral	‘+14 −100 −4’	1557	0.815443	0.434038	30.525	−4.85	<0.0001
Anterior Cingulate Gyrus	‘+0 +42 −4’	444	0.090492	−0.29264	52.76195	−7.39	0.000301

**Notes:** Percentage change between pre- and during-fALFF measures was calculated using the absolute values of each measure with the following equation: ((dur − pre)/(dur + pre)) × 100.

**Table 2:** Simultaneous tDCS effects in multiple sclerosis patients at baseline.

Main Brain Regions	Coordinates (x, y, z)	Voxels	Pre-tDCS (Mean; a.u.)	Dur-tDCS (Mean; a.u.)	Percentage Change (%)	T-Stat [19]	P-FDR
**Increased fALFF**							
Cuneal Cortex Bilateral	‘−16 −100 +18’	1172	0.429658	0.866538	33.70483	4.88	<0.0001
Superior Parietal Lobule Left	‘−32 −32 +32’	433	−0.59321	−0.15992	57.5313	7.28	0.000838
Lateral Occipital Cortex Left	‘−36 −64 +32’	332	0.106048	0.485274	64.13187	8.92	0.005065
Caudate Right	‘+16 + 28 −2’	265	−0.51554	−0.09985	67.5479	6.57	0.018587
Inferior Temporal Gyrus Right	‘+38 −62 −4’	230	−0.32848	0.089951	57.0058	5.96	0.035937
Superior Lateral Occipital Cortex Left	‘−30 −78 +2’	221	−0.21563	0.194086	5.25928	5.62	0.037842
**Decreased fALFF**							
Middle Temporal Gyrus Right	‘+68 −34 +6’	427	0.250174	−0.16139	21.571	−9.92	0.001401
Superior Frontal Gyrus LeftMiddle Frontal Gyrus Left	‘−16 −6 +76’	403	0.545782	0.092736	70.9526	−5.36	0.001401
Middle Temporal Gyrus Left	‘−58 −50 −10’	380	0.465669	0.025445	89.6379	−8.65	0.001401
Superior Frontal Gyrus RightMiddle Frontal Gyrus Right	‘+20 −6 +70’	375	0.481211	0.052951	80.1742	−6.11	0.001401
Inferior Temporal Gyrus Right	‘+54 −54 −26’	365	0.320968	−0.17819	28.6043	−7.96	0.001401
Superior Frontal Gyrus Right	‘+8 +18 +70’	234	0.410741	−0.05509	76.3488	−6.22	0.026164
Posterior Cingulate Gyrus	‘+6 −28 +28’	217	−0.22283	−0.70026	51.72135	−5.37	0.034891

**Notes:** Percentage change between pre- and during-fALFF measures was calculated using the absolute values of each measure with the following equation: ((dur − pre)/(dur + pre)) × 100.

**Table 3:** Simultaneous tDCS effects in multiple sclerosis patients at follow-up.

Main Brain Regions	Coordinates (x, y, z)	Voxels	Pre-tDCS (Mean; a.u.)	Dur-tDCS (Mean; a.u.)	Percentage Change (%)	T-Stat [19]	P-FDR
**Increased fALFF**							
Inferior Lateral Occipital Cortex Left Temporal Occipital Cortex Left	‘−40 −60 −14‘	437	0.118253	0.481962	60.59631	5.80	0.000389
Temporal Pole Right Inferior Frontal Gyrus Right	‘+52 +16 −28‘	428	−0.28435	0.110447	44.0492	9.57	0.000389
Insular Cortex Right Putamen Right Amygdala Right	‘+40 +16 −8‘	307	−0.56942	−0.18799	50.3599	12.81	0.004547
Cerebellum Right	‘+16 −86 −32‘	267	−0.02321	0.323126	86.59579	9.31	0.009520
Occipital Pole Right	‘−10 −98 +4‘	236	0.434013	0.809296	30.18424	5.04	0.016270
Inferior Frontal Gyrus Left	‘−54 +30 −2‘	232	0.093637	0.474607	67.04334	6.18	0.016270
Hippocampus Right	‘+16 −22 −40‘	225	−0.8762	−0.46149	31.0015	9.05	0.016928
Middle Frontal Gyrus Right Inferior Frontal Gyrus Right	‘+48 +46 +16 ‘	209	−0.19298	0.21055	4.355137	6.51	0.023246
Temporal Pole Left Insular Cortex Left	‘−32 +20 −10‘	191	−0.53496	−0.13044	60.7937	7.60	0.034792
**Decreased fALFF**							
Cuneal Cortex Right	‘+24 −74 +42‘	627	1.17035	0.827826	−17.1418	−7.27	0.000012
Superior Frontal Gyrus Left Middle Frontal Gyrus Left	‘−22 +34 +44‘	309	0.269823	−0.13118	−34.5747	−6.27	0.006600
Lateral Occipital Cortex Right Angular Gyrus Right	‘+46 −76 +36‘	283	0.92414	0.572967	−23.4568	−6.09	0.008517
Precuneus Cortex Superior Parietal Lobule Right	‘−10 −52 +44‘	254	0.732296	0.385454	−31.0304	−8.81	0.012849
Posterior Cingulate Gyrus	‘−2 −22 +34‘	248	0.427365	0.071968	−71.1742	−8.29	0.012849

**Notes:** Percentage change between pre- and during-fALFF measures was calculated using the absolute values of each measure with the following equation: ((dur − pre)/(dur + pre)) × 100.

**Table 4:** Cumulative tDCS effects in multiple sclerosis.

Main Brain Regions	Coordinates (x, y, z)	Voxels	Baseline (Mean; a.u.)	Follow Up (Mean; a.u.)	Percentage Change (%)	T-Stat [38]	P-FDR
**PRE-tDCS**							
Precuneus Cortex Right Cuneal Cortex Bilateral	‘−6 −86 +24’	498	0.457145	1.012112	37.77192	4.46	0.004400
Lingual Gyrus Right Occipital Fusiform Gyrus Right Inferior Lateral Occipital Cortex Right	‘+32 −66 −6’	346	−0.17168	0.367106	36.27264	4.78	0.029954
Post and Precentral Gyri Right	‘+26 −30 +44’	327	−0.16212	0.308116	31.04644	7.65	0.029954
Frontal Pole Right Middle Frontal Gyrus Right Inferior Frontal Gyrus Right	‘+24 −74 +42’	385	0.265931	−0.38734	18.58469	−6.45	0.030771
**DUR-tDCS**							
Frontal Pole Left Superior Frontal Gyrus Left	‘−38 +54 +24’	370	0.297546	−0.43713	18.99966	−5.55	0.024952
Middle Frontal Gyrus Left Inferior Frontal Gyrus Left	‘−48 +24 +44’	362	0.145045	−0.54301	57.83924	−4.63	0.024952

**Notes:** Percentage change between baseline and follow-up fALFF measures was calculated using the absolute values of each measure with the following equation: ((follow up − baseline)/(follow up + baseline)) × 100.

**Table 5:** Comparison of HC and MS fALFF measures in pre-tDCS and during-tDCS.

Main Brain Regions	Coordinates (x, y, z)	Voxels	HC (Mean; a.u.)	MS (Mean; a.u.)	Relative Difference (%)	T-Stat (46)	P-FDR
**PRE-tDCS**							
Anterior Cingulate Gyrus Thalamus Bilateral	‘+12 −30 +10‘	3346	0.159017278	−0.463121198	48.880423	6.13	<0.0001
Insular Cortex Right Frontal Operculum Cortex Right	‘−42 +10 −8‘	1231	0.097547268	−0.520021303	68.409251	5.10	<0.0001
Insular Cortex Left Inferior Frontal Gyrus Left	‘+46 +20 +0‘	1158	0.097244857	−0.492867159	67.041899	6.03	<0.0001
Posterior Inferior Temporal Gyrus Left	‘+14 −76 +30‘	518	1.026043849	0.457400933	38.3325974	5.63	0.0006
Anterior Parahippocampal Gyrus Right	‘+18 −94 +2‘	494	1.027096386	0.433568696	40.6340712	4.79	0.0007
Occipital Pole Right	‘−2 +8 −18‘	409	−0.290480644	−0.990572217	54.649702	3.42	0.0026
Insular Cortex Left Heschl’s Gyrus Left	‘+54 −58 +44‘	336	0.747053189	0.154194933	65.7819131	4.94	0.00857
Inferior Temporal Gyrus Right	‘−54 −20 +22‘	323	0.719479415	0.156920041	64.189836	5.53	0.0096
Precuneous Cortex Cuneal Cortex	‘+26 −58 −12‘	252	0.441386577	−0.098704592	63.4489146	4.38	0.0351
**DUR-tDCS**							
Posterior Inferior Temporal Gyrus Left	‘−56 −46 −28‘	1553	−0.6197054	−0.1267058	66.0493289	−8.43	<0.0001
Anterior Middle Temporal Gyrus Right	‘+38 −30 −14‘	830	−0.7224977	−0.2598023	47.1032603	−7.71	<0.0001
Superior Lateral Occipital Cortex Right	‘+48 −32 +32‘	581	−0.1409356	0.37347333	45.204852	−7.16	<0.0001
Cerebellum	‘−20 −40 −58‘	427	−0.682366	−0.044775	87.6846512	−3.60	<0.0001
Paracingulate Gyrus Left	‘−10 +24 +14‘	406	−0.8173234	−0.3373407	41.5690316	−6.75	<0.0001
Supplementary Motor Cortex Right	‘+10 +16 +52‘	377	−0.3995375	0.05429481	76.0727417	−8.13	<0.0001
Middle Temporal Gyrus Right	‘+54 −48 −6‘	332	0.0917993	0.65752227	75.498024	−6.49	<0.0001
Putamen Left	‘−24 +0 +12‘	281	−0.7584529	−0.3172187	41.019413	−6.27	0.0002
Precuneus Cortex	‘−8 −60 +40‘	263	0.19427928	0.7033319	56.71193	−5.66	0.0003
Cerebellum	‘−24 −44 −20‘	262	−0.3793384	0.06941254	69.0641142	−5.59	0.0003
Parietal Operculum Cortex Left	‘−42 −36 +24‘	254	−0.235471	0.30670603	13.138704	−6.01	0.0004

**Notes:** Positive t-statistics mean measures of fALFF in healthy controls (HCs) are greater than in multiple sclerosis (MS) patients. For negative t-statistics, however, mean measures are greater in MS compared to HCs. The relative difference between HC and MS fALFF measures was calculated using the absolute values of each measure with the following equation: ((MS − HC)/(MC + HC)) × 100. Data adjusted for age.

## Data Availability

Datasets supporting the conclusions of the present study will be made available only upon request to the corresponding author.

## References

[1] Bikson M., Grossman P., Thomas C., Zannou A.L., Jiang J., Adnan T., Mourdoukoutas A.P., Kronberg G., Truong D., Boggio P. (2016). Safety of Transcranial Direct Current Stimulation: Evidence Based Update 2016. Brain Stimul..

[2] Dedoncker J., Baeken C., De Raedt R., Vanderhasselt M.A. (2021). Combined transcranial direct current stimulation and psychological interventions: State of the art and promising perspectives for clinical psychology. Biol. Psychol..

[3] Nitsche M.A., Cohen L.G., Wassermann E.M., Priori A., Lang N., Antal A., Paulus W., Hummel F., Boggio P.S., Fregni F. (2008). Transcranial direct current stimulation: State of the art 2008. Brain Stimul..

[4] Fregni F., El-Hagrassy M.M., Pacheco-Barrios K., Carvalho S., Leite J., Simis M., Brunelin J., Nakamura-Palacios E.M., Marangolo P., Venkatasubramanian G. (2021). Evidence-Based Guidelines and Secondary Meta-Analysis for the Use of Transcranial Direct Current Stimulation in Neurological and Psychiatric Disorders. Int. J. Neuropsychopharmacol..

[5] Breitling C., Zaehle T., Dannhauer M., Bonath B., Tegelbeckers J., Flechtner H.H., Krauel K. (2016). Improving Interference Control in ADHD Patients with Transcranial Direct Current Stimulation (tDCS). Front. Cell. Neurosci..

[6] O’Shea J., Boudrias M.H., Stagg C.J., Bachtiar V., Kischka U., Blicher J.U., Johansen-Berg H. (2014). Predicting behavioural response to TDCS in chronic motor stroke. Neuroimage.

[7] Ishikuro K., Dougu N., Nukui T., Yamamoto M., Nakatsuji Y., Kuroda S., Matsushita I., Nishimaru H., Araujo M.F.P., Nishijo H. (2018). Effects of Transcranial Direct Current Stimulation (tDCS) Over the Frontal Polar Area on Motor and Executive Functions in Parkinson’s Disease; A Pilot Study. Front. Aging Neurosci..

[8] Eilam-Stock T., George A., Charvet L.E. (2021). Cognitive Telerehabilitation with Transcranial Direct Current Stimulation Improves Cognitive and Emotional Functioning Following a Traumatic Brain Injury: A Case Study. Arch. Clin. Neuropsychol..

[9] Charvet L., Shaw M., Dobbs B., Frontario A., Sherman K., Bikson M., Datta A., Krupp L., Zeinapour E., Kasschau M. (2018). Remotely Supervised Transcranial Direct Current Stimulation Increases the Benefit of At-Home Cognitive Training in Multiple Sclerosis. Neuromodulation.

[10] Agarwal S., Pawlak N., Cucca A., Sharma K., Dobbs B., Shaw M., Charvet L., Biagioni M. (2018). Remotely-supervised transcranial direct current stimulation paired with cognitive training in Parkinson’s disease: An open-label study. J. Clin. Neurosci..

[11] DeLuca G.C., Williams K., Evangelou N., Ebers G.C., Esiri M.M. (2006). The contribution of demyelination to axonal loss in multiple sclerosis. Brain.

[12] Tullman M.J. (2013). Overview of the epidemiology, diagnosis, and disease progression associated with multiple sclerosis. Am. J. Manag. Care.

[13] Ford H. (2020). Clinical presentation and diagnosis of multiple sclerosis. Clin. Med..

[14] Charvet L.E., Dobbs B., Shaw M.T., Bikson M., Datta A., Krupp L.B. (2018). Remotely supervised transcranial direct current stimulation for the treatment of fatigue in multiple sclerosis: Results from a randomized, sham-controlled trial. Mult. Scler..

[15] Pilloni G., Choi C., Shaw M.T., Coghe G., Krupp L., Moffat M., Cocco E., Pau M., Charvet L. (2020). Walking in multiple sclerosis improves with tDCS: A randomized, double-blind, sham-controlled study. Ann. Clin. Transl. Neurol..

[16] Simani L., Roozbeh M., Shojaei M., Ramezani M., Roozbeh M., Gharehgozli K., Rostami M. (2022). The effectiveness of anodal tDCS and cognitive training on cognitive functions in multiple sclerosis; a randomized, double-blind, parallel-group study. Mult. Scler. Relat. Disord..

[17] Bolognini N., Pascual-Leone A., Fregni F. (2009). Using non-invasive brain stimulation to augment motor training-induced plasticity. J. Neuroeng. Rehabil..

[18] Williams J.A., Pascual-Leone A., Fregni F. (2010). Interhemispheric modulation induced by cortical stimulation and motor training. Phys. Ther..

[19] Ke Y., Liu S., Chen L., Wang X., Ming D. (2023). Lasting enhancements in neural efficiency by multi-session transcranial direct current stimulation during working memory training. NPJ Sci. Learn..

[20] Muccio M., Walton Masters L., Pilloni G., He P., Krupp L., Datta A., Bikson M., Charvet L., Ge Y. (2022). Cerebral metabolic rate of oxygen (CMRO(2)) changes measured with simultaneous tDCS-MRI in healthy adults. Brain Res..

[21] Jamil A., Batsikadze G., Kuo H.I., Meesen R.L.J., Dechent P., Paulus W., Nitsche M.A. (2020). Current intensity- and polarity-specific online and aftereffects of transcranial direct current stimulation: An fMRI study. Hum. Brain Mapp..

[22] Muccio M., Pilloni G., Walton Masters L., He P., Krupp L., Datta A., Bikson M., Charvet L., Ge Y. (2024). Simultaneous and cumulative effects of tDCS on cerebral metabolic rate of oxygen in multiple sclerosis. Front. Hum. Neurosci..

[23] Im J.J., Jeong H., Bikson M., Woods A.J., Unal G., Oh J.K., Na S., Park J.S., Knotkova H., Song I.U. (2019). Effects of 6-month at-home transcranial direct current stimulation on cognition and cerebral glucose metabolism in Alzheimer’s disease. Brain Stimul..

[24] Ulam F., Shelton C., Richards L., Davis L., Hunter B., Fregni F., Higgins K. (2015). Cumulative effects of transcranial direct current stimulation on EEG oscillations and attention/working memory during subacute neurorehabilitation of traumatic brain injury. Clin. Neurophysiol..

[25] Alonzo A., Brassil J., Taylor J.L., Martin D., Loo C.K. (2012). Daily transcranial direct current stimulation (tDCS) leads to greater increases in cortical excitability than second daily transcranial direct current stimulation. Brain Stimul..

[26] Ho K.A., Taylor J.L., Chew T., Galvez V., Alonzo A., Bai S., Dokos S., Loo C.K. (2016). The Effect of Transcranial Direct Current Stimulation (tDCS) Electrode Size and Current Intensity on Motor Cortical Excitability: Evidence From Single and Repeated Sessions. Brain Stimul..

[27] Meyer B., Mann C., Gotz M., Gerlicher A., Saase V., Yuen K.S.L., Aedo-Jury F., Gonzalez-Escamilla G., Stroh A., Kalisch R. (2019). Increased Neural Activity in Mesostriatal Regions after Prefrontal Transcranial Direct Current Stimulation and l-DOPA Administration. J. Neurosci..

[28] He F., Li Y., Li C., Fan L., Liu T., Wang J. (2021). Repeated anodal high-definition transcranial direct current stimulation over the left dorsolateral prefrontal cortex in mild cognitive impairment patients increased regional homogeneity in multiple brain regions. PLoS ONE.

[29] Tu Y., Cao J., Guler S., Chai-Zhang T., Camprodon J.A., Vangel M., Gollub R.L., Dougherty D.D., Kong J. (2021). Perturbing fMRI brain dynamics using transcranial direct current stimulation. Neuroimage.

[30] Sankarasubramanian V., Cunningham D.A., Potter-Baker K.A., Beall E.B., Roelle S.M., Varnerin N.M., Machado A.G., Jones S.E., Lowe M.J., Plow E.B. (2017). Transcranial Direct Current Stimulation Targeting Primary Motor Versus Dorsolateral Prefrontal Cortices: Proof-of-Concept Study Investigating Functional Connectivity of Thalamocortical Networks Specific to Sensory-Affective Information Processing. Brain Connect..

[31] Kim K., Sherwood M.S., McIntire L.K., McKinley R.A., Ranganath C. (2021). Transcranial Direct Current Stimulation Modulates Connectivity of Left Dorsolateral Prefrontal Cortex with Distributed Cortical Networks. J. Cogn. Neurosci..

[32] Nissim N.R., O’Shea A., Indahlastari A., Kraft J.N., von Mering O., Aksu S., Porges E., Cohen R., Woods A.J. (2019). Effects of Transcranial Direct Current Stimulation Paired With Cognitive Training on Functional Connectivity of the Working Memory Network in Older Adults. Front. Aging Neurosci..

[33] Gao Y., Wang X., Xiong Z., Ren H., Liu R., Wei Y., Li D. (2021). Abnormal Fractional Amplitude of Low-Frequency Fluctuation as a Potential Imaging Biomarker for First-Episode Major Depressive Disorder: A Resting-State fMRI Study and Support Vector Machine Analysis. Front. Neurol..

[34] Sarappa C., Salvatore E., Filla A., Cocozza S., Russo C.V., Sacca F., Brunetti A., De Michele G., Quarantelli M. (2017). Functional MRI signal fluctuations highlight altered resting brain activity in Huntington’s disease. Brain Imaging Behav..

[35] Hu S., Chao H.H., Zhang S., Ide J.S., Li C.S. (2014). Changes in cerebral morphometry and amplitude of low-frequency fluctuations of BOLD signals during healthy aging: Correlation with inhibitory control. Brain Struct. Funct..

[36] Leaver A.M., Gonzalez S., Vasavada M., Kubicki A., Jog M., Wang D.J.J., Woods R.P., Espinoza R., Gollan J., Parrish T. (2022). Modulation of brain networks during MR-compatible transcranial direct current stimulation. Neuroimage.

[37] Paracampo R., Montemurro M., de Vega M., Avenanti A. (2018). Primary motor cortex crucial for action prediction: A tDCS study. Cortex.

[38] Whitfield-Gabrieli S., Nieto-Castanon A. (2012). Conn: A functional connectivity toolbox for correlated and anticorrelated brain networks. Brain Connect..

[39] Nieto-Castanon A. (2020). FMRI minimal preprocessing pipeline. Handbook of Functional Connectivity Magnetic Resonance Imaging Methods in CONN.

[40] Karavallil Achuthan S., Coburn K.L., Beckerson M.E., Kana R.K. (2023). Amplitude of low frequency fluctuations during resting state fMRI in autistic children. Autism Res..

[41] Ashburner J., Friston K.J. (2005). Unified segmentation. Neuroimage.

[42] Ashburner J. (2007). A fast diffeomorphic image registration algorithm. Neuroimage.

[43] Nieto-Castanon A. (2020). FMRI denoising pipeline. Handbook of Functional Connectivity Magnetic Resonance Imaging Methods in CONN.

[44] Zou Q.H., Zhu C.Z., Yang Y., Zuo X.N., Long X.Y., Cao Q.J., Wang Y.F., Zang Y.F. (2008). An improved approach to detection of amplitude of low-frequency fluctuation (ALFF) for resting-state fMRI: Fractional ALFF. J. Neurosci. Methods.

[45] Yang H., Long X.Y., Yang Y., Yan H., Zhu C.Z., Zhou X.P., Zang Y.F., Gong Q.Y. (2007). Amplitude of low frequency fluctuation within visual areas revealed by resting-state functional MRI. Neuroimage.

[46] Chumbley J., Worsley K., Flandin G., Friston K. (2010). Topological FDR for neuroimaging. Neuroimage.

[47] Kuo M.F., Chen P.S., Nitsche M.A. (2017). The application of tDCS for the treatment of psychiatric diseases. Int. Rev. Psychiatry.

[48] Floel A. (2014). tDCS-enhanced motor and cognitive function in neurological diseases. Neuroimage.

[49] Sanches C., Stengel C., Godard J., Mertz J., Teichmann M., Migliaccio R., Valero-Cabre A. (2020). Past, Present, and Future of Non-invasive Brain Stimulation Approaches to Treat Cognitive Impairment in Neurodegenerative Diseases: Time for a Comprehensive Critical Review. Front. Aging Neurosci..

[50] Ferrucci R., Vergari M., Cogiamanian F., Bocci T., Ciocca M., Tomasini E., De Riz M., Scarpini E., Priori A. (2014). Transcranial direct current stimulation (tDCS) for fatigue in multiple sclerosis. NeuroRehabilitation.

[51] Callan D.E., Falcone B., Wada A., Parasuraman R. (2016). Simultaneous tDCS-fMRI Identifies Resting State Networks Correlated with Visual Search Enhancement. Front. Hum. Neurosci..

[52] Mancuso L.E., Ilieva I.P., Hamilton R.H., Farah M.J. (2016). Does Transcranial Direct Current Stimulation Improve Healthy Working Memory?: A Meta-analytic Review. J. Cogn. Neurosci..

[53] Sela T., Lavidor M., Knotkova H., Rasche D. (2015). Enhancement of Sensory and Cognitive Functions in Healthy Subjects. Textbook of Neuromodulation.

[54] Li A., Emond L., Nattie E. (2008). Brainstem catecholaminergic neurons modulate both respiratory and cardiovascular function. Adv. Exp. Med. Biol..

[55] Benarroch E.E. (2018). Brainstem integration of arousal, sleep, cardiovascular, and respiratory control. Neurology.

[56] Mayberg H.S. (1997). Limbic-cortical dysregulation: A proposed model of depression. J. Neuropsychiatry Clin. Neurosci..

[57] Fu C.H., Williams S.C., Cleare A.J., Brammer M.J., Walsh N.D., Kim J., Andrew C.M., Pich E.M., Williams P.M., Reed L.J. (2004). Attenuation of the neural response to sad faces in major depression by antidepressant treatment: A prospective, event-related functional magnetic resonance imaging study. Arch. Gen. Psychiatry.

[58] Anand A., Li Y., Wang Y., Wu J., Gao S., Bukhari L., Mathews V.P., Kalnin A., Lowe M.J. (2005). Activity and connectivity of brain mood regulating circuit in depression: A functional magnetic resonance study. Biol. Psychiatry.

[59] Chhatbar P.Y., Kautz S.A., Takacs I., Rowland N.C., Revuelta G.J., George M.S., Bikson M., Feng W. (2018). Evidence of transcranial direct current stimulation-generated electric fields at subthalamic level in human brain in vivo. Brain Stimul..

[60] Gomez-Tames J., Asai A., Hirata A. (2020). Significant group-level hotspots found in deep brain regions during transcranial direct current stimulation (tDCS): A computational analysis of electric fields. Clin. Neurophysiol..

[61] MacKenzie-Graham A., Kurth F., Itoh Y., Wang H.J., Montag M.J., Elashoff R., Voskuhl R.R. (2016). Disability-Specific Atlases of Gray Matter Loss in Relapsing-Remitting Multiple Sclerosis. JAMA Neurol..

[62] Colato E., Stutters J., Tur C., Narayanan S., Arnold D.L., Gandini Wheeler-Kingshott C.A.M., Barkhof F., Ciccarelli O., Chard D.T., Eshaghi A. (2021). Predicting disability progression and cognitive worsening in multiple sclerosis using patterns of grey matter volumes. J. Neurol. Neurosurg. Psychiatry.

[63] Keeser D., Meindl T., Bor J., Palm U., Pogarell O., Mulert C., Brunelin J., Moller H.J., Reiser M., Padberg F. (2011). Prefrontal transcranial direct current stimulation changes connectivity of resting-state networks during fMRI. J. Neurosci..

[64] Bouchard A.E., Renauld E., Fecteau S. (2023). Changes in resting-state functional MRI connectivity during and after transcranial direct current stimulation in healthy adults. Front. Hum. Neurosci..

[65] Kasahara K., Tanaka S., Hanakawa T., Senoo A., Honda M. (2013). Lateralization of activity in the parietal cortex predicts the effectiveness of bilateral transcranial direct current stimulation on performance of a mental calculation task. Neurosci. Lett..

[66] Rosso C., Valabregue R., Arbizu C., Ferrieux S., Vargas P., Humbert F., Attal Y., Messe A., Zavanone C., Meunier S. (2014). Connectivity between right inferior frontal gyrus and supplementary motor area predicts after-effects of right frontal cathodal tDCS on picture naming speed. Brain Stimul..

[67] Cavaliere C., Aiello M., Di Perri C., Amico E., Martial C., Thibaut A., Laureys S., Soddu A. (2016). Functional Connectivity Substrates for tDCS Response in Minimally Conscious State Patients. Front. Cell. Neurosci..

[68] Greinacher R., Buhot L., Moller L., Learmonth G. (2019). The time course of ineffective sham-blinding during low-intensity (1 mA) transcranial direct current stimulation. Eur. J. Neurosci..

[69] Turi Z., Csifcsak G., Boayue N.M., Aslaksen P., Antal A., Paulus W., Groot J., Hawkins G.E., Forstmann B., Opitz A. (2019). Blinding is compromised for transcranial direct current stimulation at 1 mA for 20 min in young healthy adults. Eur. J. Neurosci..

[70] Fonteneau C., Mondino M., Arns M., Baeken C., Bikson M., Brunoni A.R., Burke M.J., Neuvonen T., Padberg F., Pascual-Leone A. (2019). Sham tDCS: A hidden source of variability? Reflections for further blinded, controlled trials. Brain Stimul..

[71] De Smet S., Nikolin S., Moffa A., Suen P., Vanderhasselt M.A., Brunoni A.R., Razza L.B. (2021). Determinants of sham response in tDCS depression trials: A systematic review and meta-analysis. Prog. Neuropsychopharmacol. Biol. Psychiatry.

[72] Loo C.K., Husain M.M., McDonald W.M., Aaronson S., O’Reardon J.P., Alonzo A., Weickert C.S., Martin D.M., McClintock S.M., Mohan A. (2018). International randomized-controlled trial of transcranial Direct Current Stimulation in depression. Brain Stimul..

[73] Ammann C., Lindquist M.A., Celnik P.A. (2017). Response variability of different anodal transcranial direct current stimulation intensities across multiple sessions. Brain Stimul..

[74] Chew T., Ho K.A., Loo C.K. (2015). Inter- and Intra-individual Variability in Response to Transcranial Direct Current Stimulation (tDCS) at Varying Current Intensities. Brain Stimul..

[75] Penolazzi B., Pastore M., Mondini S. (2013). Electrode montage dependent effects of transcranial direct current stimulation on semantic fluency. Behav. Brain Res..

[76] Hassanzahraee M., Nitsche M.A., Zoghi M., Jaberzadeh S. (2020). Determination of anodal tDCS duration threshold for reversal of corticospinal excitability: An investigation for induction of counter-regulatory mechanisms. Brain Stimul..

[77] Agboada D., Mosayebi-Samani M., Kuo M.F., Nitsche M.A. (2020). Induction of long-term potentiation-like plasticity in the primary motor cortex with repeated anodal transcranial direct current stimulation—Better effects with intensified protocols?. Brain Stimul..

[78] Muccio M., Chu D., Minkoff L., Kulkarni N., Damadian B., Damadian R.V., Ge Y. (2021). Upright versus supine MRI: Effects of body position on craniocervical CSF flow. Fluids Barriers CNS.

[79] Sun Z., Jiang D., Liu P., Muccio M., Li C., Cao Y., Wisniewski T.M., Lu H., Ge Y. (2022). Age-Related Tortuosity of Carotid and Vertebral Arteries: Quantitative Evaluation With MR Angiography. Front. Neurol..

[80] Li C., Buch S., Sun Z., Muccio M., Jiang L., Chen Y., Haacke E.M., Zhang J., Wisniewski T.M., Ge Y. (2024). In vivo mapping of hippocampal venous vasculature and oxygenation using susceptibility imaging at 7T. Neuroimage.

